# Editorial: Bioengineering and translational research for bone and joint diseases

**DOI:** 10.3389/fbioe.2022.969416

**Published:** 2022-08-26

**Authors:** Yun Dou, Yin Fang, Chao Zhao, Weili Fu, Dong Jiang

**Affiliations:** ^1^ Peking University Third Hospital, Beijing, China; ^2^ Nanyang Technological University, Singapore, Singapore; ^3^ University of Alabama, Tuscaloosa, AL, United States; ^4^ West China Hospital, Sichuan University, Chengdu, China

**Keywords:** biomaterials, biomimetic scaffolds, musculoskeletal disorders, regenerative medicine, bioengineering

Musculoskeletal disorders, commonly caused by sporting injuries, aging, accidents, and pathological factors, are one of the nonnegligible burdens that render severe pain and disability (Shang et al., 2022). As a long-term and costing disease, osteoarthritis occurs at a six folds higher rate, secondary to joint and bone lesions such as ligament rupture, cartilage defects, meniscus tear, and bone injuries (Snoeker et al., 2020). Different tissues show a great variation in the ability of post-injury self-repair. Due to increased angiogenesis and the capability to differentiate osteoblasts (Glowacki, 1998), bone is recognized as relatively prone to heal when lesions are small, whereas large instances remain challenging clinically and preclinically (Schemitsch, 2017). Unlike the adequation of blood supply in bone tissue, articular cartilage is an avascular, alymphatic, aneural, and hypocellular structure (Pathria et al., 2016). Chondrocytes, the predominant and well-differentiated type of cells in cartilage, cluster in the lacuna rich in water, proteoglycans, and collagens. Under natural situations, articular cartilage microstructure degenerates in pace with systemic aging, such as a loss in water volume and thinning of the calcified cartilage layer (Hoemann et al., 2012). Due to its biological properties, once injured, cartilage can hardly heal itself, rather injury progresses to the deeper layers and is finally in need of arthroplasty (Baumann et al., 2019). Current strategies, including conservative surgeries like arthroscopic debridement and chondroplasty (Chilelli et al., 2017), bone marrow stimulating therapy like microfracture (Allahabadi et al., 2021), and autologous transfer therapy such as osteochondral autograft transfer (McCormick et al., 2014) cated on the tibia plateau, are indispensable structures that facilitate load transmission, shock absorption, joint lubrication, and proprioception (Fox et al., 2015). Meniscus tears, commonly due to trauma or degenerative diseases, badly influence the mobility of knees, especially in athletes (Fox et al., 2015). With adequate vascularity only in the outer red-red zone, tears occurring in the inner white-white zone cannot self-repair (Makris et al., 2011). Tendon and ligament injuries are also health problems that cause pain and instability of the joints (Donderwinkel et al., 2022). Current therapies including ligament repairing and reconstruction, though commonly used in clinical settings, still have some limits, such as complications and imperfect biomechanics (Pang et al., 2022).

Tissue Engineering (TE), a combination of material science, engineering science, and regenerative medicine, is a booming and emerging technique in recent years (Wang et al., 2022). TE has already achieved conspicuous development based on the elaboration of mechanisms of injury and repair (Hodgkinson et al., 2022), the excavation of novel materials with potential use in regenerative medicine (Kluyskens et al., 2021), use of manufacturing products through advancing techniques like 3D printing (Hatt et al., 2001) and electrospinning (Lim, 2022), and by being applied *in vivo* to realize tissue repair. Despite these promising prospects, some limitations are still hindering its clinical application, such as cell sourcing, biological variability, biomimicry, implant integration and protection, inflammation, and immunogenicity (Kwon et al., 2019). This Research Topic explores the development of bioengineering strategies and their potential for clinical translation.

Primarily, we focus on research that uncovers mechanisms fundamental to bioengineering. Vascularity and mechanical loading are essential factors to promote osteanagenesis after injury. An interesting study by Zhou et al. explors the effect of inter-fragmentary gap size on neovascularization. Comparing rats fixed with different gap sizes after osteotomy, they observe that smaller gaps benefit neovascularization in the early stages, while larger gaps delay the occurrence of angiogenesis at a later phase. Liu et al. discuss the potential mechanism of mechanical strain effects and how they induce osteogenic differentiation of mesenchymal stem cells. By seeding bone marrow mesenchymal stem cells (BMSC) on the surface of TiO_2_ nanotube-modified titanium matrix loaded with cyclic stress, which exhibited induction of osteogenic differentiation of BMSC, their studies demonstrated that histone acetyltransferase GCN5, a member of histone acetyltransferases located in the nucleus, is upregulated when cyclic mechanical stress is loaded, hence promoting osteogenic differentiation through positively effecting the downstream Wnt/β-Catenin signaling pathway.

In addition, emerging evidence has revealed that systematic energy balance plays a role in bone mass regulation. Du et al. further demonstrate that exposure to cold temperature negatively influences bone volume (BV) in short term (14 days), whereas BV recovers to normal level in prolonged coldness exposure (28 days). To excavate the mechanism, the authors undertook further experiments, illustrating that cold exposure induces shortened canalicular length and apoptosis of osteocytes, providing evidence of the effect of bone remodeling by temperature.

Novel biomaterials are springing out with great versatility. Han et al. systemically review the opportunities and challenges of 3D bioprinting scaffolds, which can be applied in cartilage tissue engineering. They summarize inspiring bio-inks based on hydrogels like hyaluronic acid, gelatin, cellulose, alginate, and artificially synthetic materials like PEG, PLGA, PCL, and PLA, emphasizing that while excellent biological properties mean they have unlimited potential, issues such as cell cultivation and delivery are still on the way.

Graphene, an emerging biomaterial with great biocompatibility and controlled biodegradability, as well as enough biomechanical strength and outstanding atomic structure stability, has been extensively applied in bone regeneration. Cheng et al. reviewed derivatives (GDs), including graphene oxide (GO) and reduced graphene oxide (rGO), and applications of graphene as biomaterials. GDs-based scaffolds compounded with hydroxyapatite or collagen showed excellent enhancement of bone tissue regeneration, while mesoporous structure facilitates vascular growth. GDs based membranes or films like graphene hydrogels demonstrate even better biocompatibility and osteogenic differentiation ability. Moreover, Shen et al.discuss the promising future of copper-based biomaterials in their review, ranging from their antibacterial actions to applications such as synthetic material scaffolds, hydrogels, and bone cement. They observe the excellent antibacterial properties, sustainability, various bioactivities, and low cytotoxicity of copper in lowering the risk of infection when implanting biomimetic substitutes to repair large bone defects. Yang et al.concentrate on stem cell-laden hydrogel-based 3D bioprinting techniques in their review. The authors systematically discuss hydrogels, stem cells, and growth factors, which can be applied via 3D bioprinting processes, highlighting the challenges and perspectives in this field.

A novel injectable and thermoresponsive 3D hydrogel loaded with icariin was constructed by Zhu et al., demonstrating the prominent promotion of chondrogenic differentiation of BMSC through the activation of Wnt/β-catenin signaling, thereby sustaining the integrity of cartilage and alleviating the progression of osteoarthritis *in vivo*. It is an inspiring work, encouraging attention towards the potential of Traditional Chinese Medicine, excavating in depth the mechanism and its great potential.

With appropriate biomaterials, scaffolds combined with mesenchymal stem cells (MSCs) or growth factors are delicately manufactured and applied. With the assistance of 3D-printing techniques, Li et al. and Wang et al. respectively fabricated well-designed scaffolds to facilitate bone regeneration. An β-TCP/PLGA composite scaffold incorporated with bisperoxovanadium (bPTCP scaffold) provides a novel strategy to treat avascular necrosis of the femoral head (ANFH) through promoting angiogenesis and inducing autophagy and inhibiting apoptosis by activating the AKT/mTOR signaling pathway. A Ti6Al4V scaffold incorporated with BMP-2 and osteoprotegerin (OPG) targeted bone protection in osteoporosis. Liu et al. reviewed the structure and material properties as well as the evaluation aspects of porous femoral stems designed and applied for hip arthroplasty. They elaborate on the advantages and disadvantages of each design of the porous femoral stem, emphasizing widely used structures and materials as well as some important evaluating factors. In another review, Fu et al. summarize the microstructures and properties of OC and some effective tissue engineering strategies for especially well-designed scaffolds that benefit OC tissue repair.

Differentiation of multipotent stem cells (MSCs) is another challenge in regenerative medicine. Zhou et al. systematically review current techniques in promoting meniscus regeneration, focusing mainly on the sources and characteristics of MSCs. In their review, they summarize diverse kinds of MSCs, including bone marrow-derived (BMSC), synovium-derived (SMSC), adipose-derived (AMSC), and meniscus-derived (MMSC), cartilage-derived chondrogenic progenitor cells, etc. Mao et al. investigated the potential for peripheral blood-derived mesenchymal stem cells (PBMSCs) to assist meniscal reconstruction as a novel source of MSCs. Augmented with demineralized cortical bone matrix pretreated with TGF-β3, tissue-engineered PBMSCs showed significant ability in trilineage differentiation and meniscus repair, which broadened the source of MSCs in tissue engineering. In another review by Hu et al., various strategies in chondrocyte redifferentiation were detailedly discussed. The authors summarize the different cell sources for chondrocyte cultivation, influential microenvironment factors like temperature, hypoxia, 3D culture, matrix, and growth factors, as well as gene expression regulation. Yang et al. focus their review on the RGD peptide family, a sort of polypeptide with Arg-Gly-Asp sequence functioning as integrin receptors. They discuss the diverse function of RGD peptides interacting with integrin, including cell adhesion, MSCs differentiation, bone mineralization, and OA progression regulation. In their review, they also analyze the application of RGD in bone and cartilage tissue engineering, finding it sophisticated and immature to adopt. 

Clinically, matrix-assisted autologous chondrocyte transplantation (MACT) is an emerging procedure aimed at patients with large articular cartilage lesions. Li et al. evaluated the early efficacy of type I collagen-based MACT for the treatment of isolated full-thickness cartilage lesions of the knee. They demonstrated an inspiring result and significant clinical improvement was achieved, evidenced by subjective alleviation of symptoms and objective reconstruction of cartilage surface integrity 2 years post operation.

The articles included in this Research Topic comprehensively discuss important aspects surrounding tissue engineering, ranging from basic research to clinical translation. The great efforts made in these explorations shed light on the development of this thriving subject. Despite these promising results, a great amount of work still has to be achieved, and there is a long distance between the laboratory and the clinic. More research is required to elucidate the deeper mechanisms of injury and repair, excavate biomaterials with more biomimetic characteristics, and fabricate more bionic products, ultimately benefiting more patients.
